# Dietary preferences and quality of life among dialysis patients in Pune: a cross-sectional study

**DOI:** 10.1186/s40795-023-00811-z

**Published:** 2024-01-04

**Authors:** Devaki Gokhale, Sehlaa Kaskar, Ariti Bansal

**Affiliations:** Symbiosis Institute of Health Science, Symbiosis International (Deemed University), Pune, India

**Keywords:** Kidney Disease, Quality of life, Dietary preference, And hemodialysis

## Abstract

**Background:**

To assess the dietary preference and quality of life among dialysis patients in Pune.

**Method:**

This cross-sectional study was conducted among 127 dialysis patients aged 18–70 through a one-on-one interview to record data on demographic, biochemical, diet preference, and quality of life. The anthropometric and biochemical parameters were recorded with the help of patient reports from the four dialysis centers. A kidney disease quality of life questionnaire was used to assess the quality of life.

**Result:**

The mean age of the dialysis patients was (49.1 ± 12.9), comprising of males (104, 81.9%). (63, 49.6%) of the participants belonged to the normal weight category, followed by the underweight category. No association was noted between diet preference and quality of life, but a significant difference between BMI and personal appearance was observed. Serum calcium levels were highest (64.29 ± 1.0) in individuals in the high category of the effect of kidney disease compared to low serum calcium levels (20.89 ± 14.71) in the low category of the same.

**Conclusion:**

The present study found that diet preferences were affected due to diet restrictions, but their association with quality of life was not significantly proven. The mean for the disease burden was the lowest, implying poor quality of life among dialysis patients. Therefore, providing nutrition education and counseling for dialysis patients is crucial. A detailed dialysis care plan must address all patient requirements, including medication, dietary changes, modifications, and malnutrition screenings.

## Introduction

Chronic Kidney Disease is defined as abnormalities of kidney structure or function, present for > 3 months, with health implications.” It requires one of two criteria documented for > 3 months: GFR < 60 ml/min/1.73 m2 or markers of kidney damage, including albuminuria [1]. This damage occurs slowly over some time [2]. The excretory, metabolic, and endocrine functions are also shown to decline in most individuals with CKD, and the Glomerular Filtration Rate is reduced to less than 60 ml/min/1.73 m2. Dialysis treatment is initiated in CKD to filter out the accumulated waste products when glomerular filtration rate (GFR) is < 15 mL/min and symptoms like uremia, inability to control hydration status or blood pressure, or progressive deterioration in nutritional status are observed. In hemodialysis, a dialysis fluid is utilized, pushed out of the dialyzer, made up of membranes acting as filters, and the filtered blood is returned to the body. In peritoneal dialysis, a catheter is placed through the surgical method into the peritoneal cavity, and with the help of this catheter, a sterile solution is inserted into the peritoneal cavity. Peritoneal dialysis can further be characterized as Continuous Ambulatory Peritoneal Dialysis and Automated Peritoneal Dialysis. Malnutrition is commonly observed in dialysis patients, which can be associated with uremic wastes and accelerated protein catabolism. The severity of Malnutrition is based on CKD stages and is generally more commonly observed in low-income groups with advanced stages of CKD [3]. The global estimated prevalence of CKD is 13.4%, and the patients with end-stage kidney disease who require renal replacement treatment are predicted to be between 4.902 and 7.083 million worldwide. In 2010, the global prevalence of CKD staged 1–5 in individuals aged > = 20 years was 10.4% in men and 11.8% in women [4]. In India, the prevalence of CKD was observed in 9.94% of the population, with low eGFR at 5.43% and proteinuria at 6.47% [5].

Quality of life is a broader concept concerned with whether disease or impairment limits a person’s ability to fulfill a regular role (for example, whether the inability to climb stairs limits a person at work) [6]. The QOL decreases in CKD patients as the stages progress. Participants with a low income and a low hemoglobin level were regarded to have a poorer quality of life in both the physical and mental aspects [7]. The quality of life of hemodialysis patients is substantially lower than healthy population and was found to be lower in all four World Health Organization Quality of Life Questionnaire domains compared to renal transplant patients’ quality of life [8].

A study conducted among a Japanese population found that compared to non-CKD patients, in CKD individuals, the risk is common in males, advanced age, high BMI, hypertensive patients, and individuals with diabetes, a history of stroke, or heart disease [9]. Individuals with diabetic retinopathy also experience faster progression of CKD stages [10]. Electrolyte imbalances such as hypokalemia are associated with increased diuretic use, reduced Renin-angiotensin system blockade use, and malnutrition, all hamper renal functioning. The measures of dialysis adequacy, such as serum creatinine, blood urea, serum potassium, phosphorous, and quality of life, all improve significantly after a prescribed intradialytic exercise regimen [11]. Increased alcohol intake and cigarette smoking are known risk factors for the onset of CKD progression [12, 13]. A study reported that mild-spectrum CKD individuals showed increased saturated fat, salt, and protein intake. Whereas fiber, calcium, potassium, and phosphorous consumption was low [14]. The risk of CKD can be heightened with a lack of physical activity, late-night dinners, and bedtime snacks [15].

It is known that the prevalence of CKD is on the rise worldwide, increasing the risk of other organ complications like gout, anemia, secondary hyperparathyroidism, heart disease, fluid accumulation, and mortality. Minimal studies have discussed poor dietary knowledge and low-quality life, which will be the prime focus of the present study. Furthermore, it will determine the correlation between dietary knowledge and disease severity among dialysis patients.

## Methodology

### Study design and population

This cross-sectional observational study was conducted among 127 dialysis patients aged 18 to 70 years enrolled based on inclusion and exclusion criteria from four dialysis centers across the suburban region in Pune City, Maharashtra. A selective sampling technique was used, wherein the researcher selected the participants based on the inclusion and exclusion criteria of the study. This technique was used since it is cost-effective and less time-consuming. The participants included were those belonging to the age group of 18 to ≤ 70 years on hemodialysis or peritoneal dialysis. Participants were excluded if they had kidney stones, those infected with COVID-19, or were dependent on enteral or parenteral nutrition, were pregnant, lactating, or in any other active infectious state. An informed consent form was provided to the participants who voluntarily agreed to enrolment.

### Anthropometric measurements

Anthropometric indicators such as height (cm) and weight (kg) were recorded from patient reports available at dialysis centers, and BMI was calculated from a formula using kg/m2 and was classified according to WHO [16].

### Biochemical measurements

Renal function test values such as Urea (mg), Creatinine (mg), Uric acid (mg/dl), Phosphorus (mg/dl), and calcium (mg/dl) were obtained from the available participant file records. Electrolyte values were also recorded from participant file records, including Sodium (mEq/L), Potassium (mEq/L), and Chloride (mEq/L). These renal function tests help detect kidney disease, monitor the kidney’s response to treatment, and determine the progression of kidney disease.

### Kidney Disease Quality of Life (KDQOL) Questionnaire

It is a self-report measure designed for individuals suffering from kidney disease or on dialysis. It considers parameters relevant to patients with kidney disease, such as symptoms, the burden of illness, social interaction, Work status, cognitive function, and sexual function. The symptom list evaluates the extent of bother as Not at all, Somewhat, moderately, very much, or Extremely for the past 30 days. These symptoms include muscle soreness, chest pain, numbness in hands or feet, cramps, nausea or upset stomach, lack of appetite, faintness or dizziness, shortness of breath, washed out or drained, itchy skin, dry skin, and problems with access site. KDQO-L comprises 36 questions divided into five domains as follows:

1. The measure of physical and mental functioning (Questions 1–12).
2. The burden of kidney disease (questions 13–16).
3. The symptoms and problems (questions 17–28b).
4. Effects of kidney disease on daily life (questions 29–36).

The effect of kidney disease on daily life was assessed using a five-point response scale for the symptom list. The burden of kidney disease scale assessed the perception of frustration and interference of the disease in an individual’s life using a definitely true to definitely false response scale [17].

The scoring of questions was as follows:

1. For i1, i8, i17-i27, i29-i36 (Q1, Q8, Q17-Q27, Q29-Q36), where 1 = 100, 2 = 75, 3 = 50, 4 = 25, 5 = 0,
2. For i2 & i3 (Q2, Q3), where 1 = 0, 2 = 50, 3 = 100.
3. For i4-i7 (Q4-Q7), where 1 = 0, 2 = 100.
4. For i9 & i10 (Q9-Q10), where 1 = 100, 2 = 80, 3 = 60, 4 = 40, 5 = 20, 6 = 0.
5. For i11 (Q11), where 1 = 0, 2 = 20, 3 = 40, 4 = 60, 5 = 80, 6 = 100.
6. For i12-i16 (Q12-Q16), where 1 = 0, 2 = 25, 3 = 50, 4 = 75, 5 = 100.

### Kidney Disease Questionnaire (KDQ)

It is a self-made questionnaire that includes three sections comprising personal details, Kidney based knowledge, and Diet preferences.

Section 1: It comprised personal details such as age, gender, occupation, alcohol status, smoking status, family history, comorbidities, and food habits.

Section 2: It completely focused on questions testing their kidney-related knowledge.

Section 3: It included questions related to dietary preferences such as protein preference, no. of meals per day, servings of fruits and vegetables consumed, green leafy vegetable consumption, water intake, coffee or tea intake, salt preference, intake of salad dressings, ketchup or mayonnaise, consumption of (Ready to eat) RTE products, and other questions associated with diet knowledge.

This questionnaire scored from 0 to 6, where 0 was the lowest and 6 was the highest.

### Statistical analysis

Data were entered into SPSS Version 28.0. with a 95% confidence interval, i.e., a *p*-value of < 0.05 was considered significant for all tests.

The data were presented for the continuous variables as mean ± standard deviation (SD) and frequency and Percentage (%) for the categorical variables. The Chi-square test was used to determine if there was a significant association between two categorical variables. Kidney disease quality of life scores were divided into tertiles. ANOVA was performed to determine whether there was a difference in the mean of continuous variables between three or more independent groups or categories.

## Results

Table 1 represents the Socio-Demographic details of dialysis patients. The majority (104, 81.9%) of them were males, followed by females (23, 18.1%) with a mean age of (49.1 ± 12.9). Most (68, 53.5%) of the participants were unemployed, housewives (21, 16.5%), and one student (1, 0.8%). Out of 127 participants, (85, 66.9%) were non-vegetarian, and (42, 33.1%) were vegetarian. Most (82, 64.6%) of them were suffering from Hypertension, Diabetes along with Hypertension (27, 21.3%), and only Diabetes (6, 4.7%). Only (3, 2.4%) reported a family history of CKD. Alcohol intake (45, 35.4%) and smoking status (13, 10.2%) were reported among the patients.

**Table 1:** Demographic Details

Variables	N (%)
**Gender**	
Males	104 (81.9%)
Females	23 (18.1%)
**Occupation**	
Business	8 (6.3%)
Job service	16 (12.6%)
Service	13 (10.2%)
Housewife	21 (6.5%)
Unemployed	68 (53.5%)
Student	1 (0.8%)
**Family history of CKD**	
Yes	3 (2.4%)
No	124 (97.6%)
**Food habit**	
Vegetarian	42 (33.1%)
Non-vegetarian	85 (66.9%)
**Comorbidities**	
Diabetes	6 (4.7%)
Hypertension	82 (64.6%)
Diabetes and hypertension	27 (21.3%)
No	12 (9.4%)
**Alcohol status**	
Yes	45 (35.4%)
No	82 (64.6%)
**Smoking status**	
Yes	13 (10.2%)
No	114 (89.8%)
**Age (years) Mean +- SD**	49.1 +- 12.9

*Note*: SD stands for standard Deviation, and N stands for Frequency

Anthropometric and biochemical data has been depicted in Table 2, wherein the mean BMI observed was (21.77 ± 4.74) with a mean weight of (58.1 ± 1.3) and a mean height of (163.2 ± 7.4). The BMI category of the participants is denoted as per WHO classification. Most participants (63, 49.6%) belonged to the normal category, followed by the underweight category (35, 27.6%). Among the biochemical parameters, most participants had higher creatinine levels (120, 94.5%) and urea levels (108, 85.0%). Hyponatremia was observed among (25, 19.7%), Hypocalcaemia among (27, 21.3%), and Hyperkalaemia among (15, 11.8%) of the participants.

**Table 2:** Anthropometric and Biochemical data

Anthropometric indices	Mean + SD
Height (cm)	163.2 + 7.4
Weight (kg)	58.1 + 1.3
BMI (kg/m^2^)	21.77 + 4.74
**BMI category**	**N (%)**
Underweight (< 18.5 kg/m^2^)	35 (27.6%)
Normal (18.5–24.9 kg/m^2^)	63 (49.6%)
Overweight (25.9–29.9 kg/m^2^)	20 (15.7%)
Obese ( > = 30 kg/m^2^)	9 (7.1%)
**Biochemical parameters**	**Mean** + **SD**
Serum sodium (mEq/L)	1.3 + 4.0
Serum potassium (mEq/L)	4.7 + 0.7
Serum chloride (mEq/L)	1.0 + 3.5
Serum Phosphorus (mg/dl)	4.5 + 1.1
Serum calcium(mg/dl)	9.0 + 1.1
Urea (mg/dl)	65.6 + 2.8
Uric acid (mg/dl)	5.7 + 3.2
Creatinine (mg/dl)	6.6 + 3.3

*Note*: SD stands for standard Deviation, and N stands for Frequency

Tables 3 and 4 describe the domains of KDQOL, such as physical and mental health, symptom list of kidney disease, the effect of kidney disease, and the burden of kidney disease. For general health, most patients had difficulty accomplishing their daily tasks (119, 93.7%), and (110, 86.6%) had impaired daily or work-related activities. Patient’s mental health was also affected, as (121, 95.3%) and (107, 84.3%) of patients had problems accomplishing their daily tasks and failed to do other activities mentally. An equal distribution of patients was observed associated with pain interfering with everyday tasks, i.e., (33, 26%) each for “quite a bit” and “extreme” parameters for assessing pain. The burden of kidney diseases, such as interference of disease, time spent dealing with disease, frustration, and burden on family, was experienced by most patients, i.e., (112, 88.2%), (108, 85%), (90, 70.9%), and (97, 76.4%), respectively. The symptoms and effects of kidney disease experienced majorly by the patients were itchy skin, dry skin, fluid restriction, dietary restriction, ability to work around the house, ability to travel, being dependent on others, stress caused by the disease, and personal appearance.

**Table 3:** KDQOL 1 (Physical and Mental Health)

Variables	N (%)
**General Health**	
Excellent	0
Very good	0
Good	28 (22.0%)
Fair	71 (55.9%)
Poor	28 (22.0%)
**Moderate Activities**	
Yes, it limited a lot	50 (39.4%)
Yes, limited a little	57 (44.9%)
No, not limited at all	20 (15.7%)
**Climbing several flights of stairs**	
Yes, it limited a lot	54 (42.5%)
Yes, limited a little	60 (47.2%)
No, not limited at all	13 (10.2%)
**Problems with work/ daily activities (Physical Health)**	
**Accomplished less than you would like**	
Yes	119 (93.7%)
No	8 (6.3%)
**Limited to work/Daily activities**	
Yes	110 (86.6%)
No	17 (13.4%)
**Problems with work/ daily activities (Emotional Health)**	
**Accomplished less than you would like**	
Yes	121 (95.3%)
No	6 (4.7%)
**Didn’t do work/ other activities carefully**	
Yes	107 (84.3%)
No	20 (15.7%)
**Pain Interfered with Normal work**	
Not at all	4 (3.1%)
A little bit	28 (22.0%)
Moderately	29 (22.8%)
Quite a bit	33 (26.0%)
Extremely	33 (26.0%)
**Felt calm & peaceful**	
All of the time	2 (1.6%)
Most of the time	12 (9.4%)
A good bit of the time	13 (10.2%)
Some of the time	30 (23.6%)
A little of the time	43 (33.9%)
None of the time	27 (21.3%)
**Energy in body**	
All of the time	3 (2.4%)
Most of the time	3 (2.4%)

*Note*: N stands for Frequency

**Table 4:** KDQOL 2 (symptoms, effect of kidney disease, and burden of kidney disease)

The burden of kidney disease					
Variables	Definitely True N (%)	Mostly True N (%)	Don’t Know N (%)	Mostly false N (%)	Definitely false N (%)
Kidney Disease interfered too much with life	112 (88.2%)	12 (9.4%)	0	0	3 (2.4%)
Time spent dealing with kidney disease	108 (85.0%)	15 (11.8%)	1 (0.8%)	0	3 (2.4%)
Frustrated dealing with kidney disease	90 (70.9%)	22 (17.3%)	3 (2.4%)	8 (6.3%)	4 (3.1%)
The burden on my family	97 (76.4%)	8 (6.3%)	3 (2.4%)	7 (5.5%)	12. (9.4%)
**Symptoms and effects of kidney disease on daily life**					
**Variables**	**Not at all bothered N (%)**	**Somewhat bothered N (%)**	**Moderately bothered** **N (%)**	**Very much bothered N (%)**	**Extremely bothered N (%)**
Soreness in muscles	76 (59.8%)	29 (22.8%)	16 (12.6%)	4 (3.1%)	2 (1.6%)
Chest pain	77 (60.6%)	22 (17.3%)	24 (18.9%)	2 (1.6%)	2 (1.6%)
Cramps	30 (23.6%)	26 (20.5%)	40 (31.5%)	24 (18.9%)	7 (5.5%)
Itchy skin	44 (34.6%)	6 (4.7%)	14 (11.0%)	27 (21.3%)	36 (28.3%)
Dry skin	33 (26.0%)	6 (4.7%)	11 (8.7%)	27 (21.3%)	50 (39.4%)
Shortness of breath	76 (59.8%)	26 (20.5%)	16 (12.6%)	7 (5.5%)	2 (1.6%)
Faintness or dizziness	84 (66.1%)	22 (17.3%)	17 (13.4%)	4 (3.1%)	0
Lack of appetite	47 (37.0%)	34 (26.8%)	21 (16.5%)	15 (11.8%)	10 (7.9%)
Washed out or drained	91 (71.7%)	19 (15.0%)	11 (8.7%)	6 (4.7%)	0
Numbness in hands or feet	65 (51.2%)	21 (16.5%)	27 (21.3%)	13 (10.2%)	1 (0.8%)
Nausea or upset stomach	57 (44.9%)	18 (14.2%)	16 (12.6%)	18 (14.2%)	18 (14.2%)
(Haemodialysis patient only) Problems with your access site	54 (42.5%)	46 (36.2%)	18 (14.2%)	8 (6.3%)	1 (0.8%)
Fluid restriction	10 (7.9%)	4 (3.1%)	7 (5.5%)	21 (16.5%)	85 (66.9%)
Dietary restriction	10 (7.9%)	3 (2.4%)	6 (4.7%)	29 (22.8%)	79 (62.2%)
Ability to work around the house	8 (6.3%)	7 (5.5%)	14 (11.0%)	30 (23.6%)	68 (53.5%)
Ability to travel	7 (5.5%)	2 (1.6%)	6 (4.7%)	23 (18.1%)	89 (70.1%)
Being dependent on doctors and other medical staff	15 (11.8%)	8 (6.3%)	16 (12.6%)	20 (15.7%)	68 (53.5%)
Stress or worries caused by kidney disease	29 (22.8%)	12 (9.4%)	16 (12.6%)	23 (18.1%)	47 (37.0%)
Personal appearance	47 (37.0%)	7 (5.5%)	15 (11.8%)	23 (18.1%)	35 (27.6%)

*Note*: N stands for Frequency

In Table 5, the KDQOL scores have been highlighted. The scores for all five domains can be interpreted as higher mean values indicating favorable health status and better quality of life. Low mean values, on the other hand, indicate less favorable health status and poor quality of life. The mean value (71.8 ± 12.1) for the symptom/ problem list indicates better quality of life. In contrast, for the burden of kidney disease, the lowest mean value (10.0 ± 17.8) shows a less favorable health status and poor quality of life. For other domains, such as physical health (32.9 ± 7.9), mental health (34.3 ± 7.8), and the effect of kidney disease on daily life (27.0 ± 18.0) does indicate that do have some negative impact on quality of life, though the mean values are not very high.

**Table 5:** KDQOL scores

Scale (number of items in scale)	Mean + SD	Poor N (%)	Average N (%)	Good N (%)
Symptoms/problem list (12)	71.8 + 12.1	42 (33.1%)	33 (26.0%)	52 (40.9%)
Effects of kidney disease (8)	27.0 + 18.0	33 (26.0%)	47 (37.0%)	47 (37.0%)
The burden of kidney disease (4)	10.0 + 17.8	67 (52.8%)	0	60 (47.2%)
SF-12 Physical Health Composite	32.9 + 7.9	40 (31.5%)	39 (30.7%)	48 (37.8%)
SF-12 Mental Health Composite	34.3 + 7.8	39 (30.7%)	44 (34.6%)	44 (34.6%)

*Note*: SD stands for standard Deviation, and N stands for Frequency

The association of diet preferences with patients’ quality of life has been explained in Table 6. However, no significant association was between the two. It can be interpreted from the table that fruit consumption and meal intake per day (108, 85%) of patients were highly bothered due to diet restrictions. (40, 31.5%) of patients each for daily water intake and consuming baked products experienced cramps.

**Table 6:** Association of diet preferences and KDQOL

Variables		N (%)	*X2* value	*p*-value
**Fruits consumption**	**Dietary Restriction**		0.84	0.65
	Not at all bothered	13 (10.2%)		
	Moderately bothered	6 (4.7%)		
	Extremely bothered	108 (85%)		
**Meal intake/day**	**Dietary Restriction**		4.96	0.08
	Not at all bothered	13 (10.2%)		
	Moderately bothered	6 (4.7%)		
	Extremely bothered	108 (85%)		
	**Do you feel energetic?**		3.57	0.16
	Most of the time	6 (4.7%)		
	Some of the time	54 (42.5%)		
	Little of the time	67 (52.8%)		
	**Washed out or drained** **out**		4.74	0.09
	Not at all bothered	110 (86.6%)		
	Moderately bothered	11 (8.7%)		
	Extremely bothered	6 (4.7%)		
**Water intake/day**	**Cramps**		5.83	0.21
	Not at all bothered	56 (44.1%)		
	Moderately bothered	40 (31.5%)		
	Extremely bothered	31 (24.4%)		
**Baked products**	**Cramps**		2.89	0.57
	Not at all bothered	56 (44.1%)		
	Moderately bothered	40 (31.5%)		
	Extremely bothered	31 (24.4%)		
	**Nausea or stomach** **upset**		3.21	0.52
	Not at all bothered	75 (59.1%)		
	Moderately bothered	16 (12.6%)		
	Extremely bothered	36. (28.3%)		

*Note*: N stands for Frequency, and *p-value significant at ≤ 0.05

Table 7 illustrates the two variances, first between anthropometric data and the KDQOL symptom list and the effect of the disease. For this, a significant association was observed for chest pain (*p* < 0.05), dry skin (*p* < 0.05), and personal appearance (*p* < 0.05) in relation to weight. A significant association between BMI and personal appearance can be observed (*p* < 0.05).

**Table 7:** One-Way Analysis of Variance between Anthropometric data and KDQOL symptom list & Effect of kidney disease & KDQOL domains and biochemical parameters

Variables		N (%)	Mean + SD	F value	*p*-value
Weight (Kg)	Chest pain			3.06	0.05*
	Not at all bothered	99 (78%)	59.65 + 14.17		
	Moderately bothered	24 (18.9%)	52.0 + 10.0		
	Extremely bothered	4 (3.1%)	58.0 + 11.6		
	**Dry skin**			**3.27**	0.04*
	Not at all bothered	39 (30.7%)	59.1 + 14.2		
	Moderately bothered	11 (8.7%)	48.2 + 7.8		
	Extremely bothered	77 (60.6%)	59.1 + 13.5		
	**Personal appearance**			**4.42**	0.04*
	Not at all bothered	54 (42.5%)	61.3 + 15.4		
	Moderately bothered	15 (11.8%)	61.4 + 10.4		
	Extremely bothered	58 (45.7%)	54.3 + 11.7		
**BMI** **(Kg/m2)**	**Chest pain**			2.43	0.09
	Not at all bothered	99 (78%)	22.2 + 4.9		
	Moderately bothered	24 (18.9%)	19.9 + 3.2		
	Extremely bothered	4 (3.1%)	21.0 + 3.9		
	**Personal appearance**			**2.91**	0.05*
	Not at all bothered	54 (42.5%)	22.5 + 5.3		
	Moderately bothered	15 (11.8%)	23.1 + 3.6		
	Extremely bothered	58 (45.7%)	20.7 + 4.2		
**Domains of KDQOL and Biochemical parameters**					
**Effect of kidney disease on daily life**	**Calcium levels (mg/dl)**			**4.13**	0.01**
	Low	27 (21.3%)	20.89 + 14.71		
	Normal	99 (78.0%)	28.35 + 18.35		
	High	1 (0.8%)	64.29 + 1.0		
**Physical Health**	**Uric acid levels (mg/dl)**			2.63	0.07
	Low	7 (5.5%)	28.31 + 5.88		
	Normal	102 (80.3%)	33.66 + 7.92		
	High	18 (14.2%)	30.38 + 7.67		

*Note*: N stands for Frequency, *p-value significant at ≤ 0.05, and **p value significant at ≤ 0.01

The second variance is between KDQOL domains and biochemical parameters. A significant association was observed between calcium levels and kidney disease’s effect on daily life, such as the mean calcium level was (20.89 ± 14.71) among the low categories. Although there was no significant association between uric acid level and physical health, the mean uric acid level was (28.31 ± 5.88) among the low categories.

## Discussion

The present study investigated the association between dietary preferences and quality of life among dialysis patients in Pune City. In total, 127 adults aged 18 to ≤ 70 years who were put on hemodialysis or peritoneal dialysis were recruited for this study. The study was conducted in four Dialysis centers in Pune City, Maharashtra, in 2022.

A majority (104, 81.9%) of them were males, followed by females (23, 18.1%) with mean age (49.1 ± 12.9). Most (82, 64.6%) of them were suffering from Hypertension, Diabetes along with Hypertension (27, 21.3%), and only Diabetes (6, 4.7%). The Mean BMI was observed as (21.77 ± 4.74) with a mean weight of (58.1 ± 1.3) and a mean height of (163.2 ± 7.4). The BMI category of the participants was denoted as per WHO classification. Most participants (63, 49.6%) belonged to the normal category, followed by the underweight category (35, 27.6%). Most participants had higher creatinine levels (120, 94.5%) and urea levels (108, 85.0%). Hyponatremia was observed among (25, 19.7%), Hypocalcemia among (27, 21.3%), and Hyperkalemia among (15, 11.8%) of the participants. (53, 41.7%) of individuals had poor knowledge scores, and (41, 32.3%) had average knowledge scores concerning diet and kidney disease. On the other hand, only (33, 26%) had good knowledge about kidney disease and diet. Similar studies suggest participants had low knowledge about renal diet and negative attitudes towards diet [18].

Quality of life for hemodialysis patients decreased with CKD stage progression. According to other studies, the Quality of life score in dialysis patients was considerably impacted by age and gender [19]. The mean value (71.8 ± 12.1) for the symptom/ problem list indicates a better quality of life, whereas, for the burden of kidney disease, the lowest mean value (10.0 ± 17.8) indicates less favorable health status and poor quality of life. Also, the other domains, such as physical health (32.9), mental health (34.3), and the effect of kidney disease on daily life (27.0), indicate poor quality of life. Poor health status was observed for the burden of kidney disease (52.8%). Similar studies suggested that hemodialysis patients had significantly impaired (P < 0.05) quality of life, specifically in physical, psychological, and social relationship domains [8].

This study investigated the association between dietary preferences and quality of life among dialysis patients in Pune City. So far, the studies conducted before have focused mainly on basic kidney knowledge, dietary choices, or specific to particular nutrients. From this study, we could understand the combined effect of all three variables (kidney disease, dietary preferences, and quality of life). The data collection for this study was done from 4 different dialysis centers in Pune city. Hence providing more reliable and authentic data. The first limitation of the study was subject selection bias and recall bias concerning lifestyle factors like alcohol status and smoking status, as most of the individuals were reluctant to answer accurately. Another limitation of the study was that confounding variables, such as other medical conditions, were not considered.

## Conclusion

In conclusion, our research findings revealed that while diet restrictions influenced diet preferences among dialysis patients in Pune, no significant association was established between these preferences and the overall quality of life. However, it is important to note that the burden of kidney disease significantly impacts the patient’s quality of life. Given the vital role of diet in patient recovery, it is imperative to prioritize patient education by providing comprehensive care plans that address dietary needs. Implementing counseling sessions as part of pre-dialysis care can greatly assist patients in coping with their condition and improving their overall well-being.

## Data Availability

The datasets used and analyzed during the current study are available from the corresponding author upon reasonable request.
